# Functional Modifications of Acid-Sensing Ion Channels by Ligand-Gated Chloride Channels

**DOI:** 10.1371/journal.pone.0021970

**Published:** 2011-07-18

**Authors:** Xuanmao Chen, Paul Whissell, Beverley A. Orser, John F. MacDonald

**Affiliations:** 1 Department of Physiology, University of Toronto, Toronto, Ontario, Canada; 2 Robarts Research Institute, University of Western Ontario, London, Ontario, Canada; 3 Department of Anesthesia, Sunnybrook Health Sciences Centre, University of Toronto, Toronto, Ontario, Canada; 4 Department of Anesthesia, University of Toronto, Toronto, Ontario, Canada; INSERM U901, France

## Abstract

Together, acid-sensing ion channels (ASICs) and epithelial sodium channels (ENaC) constitute the majority of voltage-independent sodium channels in mammals. ENaC is regulated by a chloride channel, the cystic fibrosis transmembrane conductance regulator (CFTR). Here we show that ASICs were reversibly inhibited by activation of GABA_A_ receptors in murine hippocampal neurons. This inhibition of ASICs required opening of the chloride channels but occurred with both outward and inward GABA_A_ receptor-mediated currents. Moreover, activation of the GABA_A_ receptors modified the pharmacological features and kinetic properties of the ASIC currents, including the time course of activation, desensitization and deactivation. Modification of ASICs by open GABA_A_ receptors was also observed in both nucleated patches and outside-out patches excised from hippocampal neurons. Interestingly, ASICs and GABA_A_ receptors interacted to regulate synaptic plasticity in CA1 hippocampal slices. The activation of glycine receptors, which are similar to GABA_A_ receptors, also modified ASICs in spinal neurons. We conclude that GABA_A_ receptors and glycine receptors modify ASICs in neurons through mechanisms that require the opening of chloride channels.

## Introduction

Extracellular protons serve as the ligand for a family of ligand-gated ion channels, acid-sensing ion channels (ASICs) [1], [2]. These channels are associated with various physiological and pathophysiological functions including regulation of synaptic plasticity [3], perception of pain [4], ischemic death of neurons [5] and the termination of seizures [6]. Recently, proteins of chicken ASIC1 were crystallized and their structures probed [7], [8]. Each ASIC subunit contains a highly conserved, cysteine-rich “thumb domain” region, which is implicated in the regulation of channel gating [7]. Surprisingly, a chloride ion is partly embedded in the thumb domain, and each trimeric channel associates with three chloride ions [7], [8]. There is limited evidence as to the functional consequences of chloride binding to ASICs although desensitization of the ASIC1a subtype is altered by changes in extracellular chloride and mutation of the chloride-binding site abolishes this regulation [9].

In the central nervous system (CNS), the most abundantly expressed chloride channels are γ-aminobutyric acid receptors (GABA_A_) and, to a lesser extent, glycine receptors. GABA_A_ receptors mediate both tonic and rapid synaptic inhibition [10]. We hypothesized that ASICs are regulated by chloride channels in CNS neurons. In the study reported here, we found that ASICs were modified by the activation of GABA_A_ receptors in hippocampal neurons. These results suggest that the proton-gated sodium channels have an intimate relationship with ligand-gated chloride channels in the CNS neurons.

## Materials and Methods

### Cell cultures

All animal experiments were carried out in accordance with guidelines approved by the University of Toronto Animal Care Committee. Cultures of dissociated spinal neurons were prepared from wild-type Swiss white mice, which were sacrificed at embryonic day 13 or 14 (E13 or E14). The whole spinal cord of each foetus was taken. For cultures of hippocampal neurons, pregnant mice were sacrificed and foetuses rapidly removed at embryonic day 17 or 18. Tissues were first dissected in cold Hanks' solution, and the spinal or hippocampal neurons were then dissociated by mechanical trituration. The dissociated neurons were plated on 35-mm culture dishes at an estimated density of less than 1×10^6^ cells/cm^2^. The cell cultures were incubated during week 1 in a minimal essential media supplemented with 10% fetal bovine serum, 10% inactivated horse serum and insulin (8 µg/ml) at 37°C in 5% carbon dioxide (cell culture chemicals from Invitrogen, Carlsbad, CA, USA). Proliferation of fibroblasts and glial cells was terminated by the addition of floxuridine at day 7 *in vitro*.

### Whole-cell voltage-clamp recordings in cultured primary spinal or hippocampal neurons

Recording pipettes were prepared from borosilicate glass capillaries (World Precision Instruments, Sarasota, FL, USA). A vertical puller (Narishige PP-83) was used to pull electrodes in two stages. Whole-cell voltage-clamp recordings were made on cultured murine spinal or hippocampal neurons 14–21 days after plating. The extracellular solution had the following composition (in mM): 140 NaCl, 2 CaCl_2_, 1 MgCl_2,_ 25 *N*-2-hydroxyethylpiperazine-*N*′-ethanesulfonic acid (HEPES), 33 glucose, 5.4 KCl and 0.0002 tetrodotoxin, with pH of 7.4 and osmolarity range from 320 to 330 mOsm. Unless otherwise indicated, the intracellular pipette solution for voltage-clamp recordings had the following composition (in mM): 140 cesium gluconate (as the main salt component), 11 ethyleneglycol-bis-(α-amino-ethyl ether) *N*,*N*′-tetra-acetic acid (EGTA), 10 HEPES, 2 MgCl_2_, 2 tetraethyl ammonium chloride (TEA-Cl), 1 CaCl_2_ and 4 K_2_ATP. The resistance ranges of pipettes filled with this solution were 2.5–4 MΩ. The acidity was adjusted to pH 7.2 with CsOH. All recordings were performed at room temperature. Unless specified otherwise, membrane potential was held at −60 mV throughout the recordings. ASIC currents were elicited by rapid application of pH 5.8 solution (HEPES buffer replaced by 2-*N*-morpholino-ethanesulfonic acid) delivered from a multi-barrelled fast perfusion system (SF-77 B, Warner Instrument Corp. Hamden, CT, USA) for a period of 0.5 to 8 s, and this procedure was repeated every minute. The perfusion rate of the solution was about 1 ml/min. Unless specified otherwise, GABA (to activate GABA_A_ receptors) was applied by perfusion for a period of 1 to 10 min. Whole-cell currents were recorded with an Axopatch-1D amplifier (Molecular Devices, Sunnyvale, CA, USA) or a Multiclamp 700B amplifier (Molecular Devices, Sunnyvale, CA, USA). Electrophysiological signals were filtered at 2 kHz and digitized at 5–10 kHz by means of a Digidata 1332A processor and/or were simultaneously filtered and digitized through a MiniDigi 1A processor; the signals were acquired online with pClamp8.2 or pClamp9.2 (Axon Instruments, Foster City, CA, USA) or/and Axoscope 9.2 (Axon Instruments, Foster City, CA, USA).

### Recording from excised nucleated patches and outside-out patches

Nucleated patches and outside-out patches were excised from cultured primary hippocampal neurons [11]. The procedure was slightly modified as previously described [12] [13]. For nucleated patches, a modest negative pressure was applied before the patch pipette was withdrawn, to attract the nucleus of the neuron to the tip of pipette. If patch formation was successful, a nucleated bulb attached to the tip of patch pipette could be seen under ×40 microscopic visualization. For both nucleated and outside-out patches, the patch pipette was placed in front of a triple-barrelled application pipette with fast solution exchange, controlled by an SF-77 B system (Warner Instrument Cooperation). A control solution (pH 7.4) or a test solution (pH 5.8) was applied (through gravity-driven flow out of the application pipette) to activate the ASICs in the patches. GABA and bicuculline were applied by perfusion, together or separately. Patches were clamped to −60 mV throughout the experiments. Electrophysiological signals were recorded with a Multiclamp 700B amplifier (Axon Instruments, Foster City, CA, USA), low-pass filtered at 2 kHz and sampled at 10 or 20 kHz.

### Field recordings

Wild-type C57/BL6 mice, 2–4 months old, were decapitated under isoflurane anaesthesia. The brains were removed and placed in oxygenated (95% O_2_, 5% CO_2_) ice-cold artificial cerebral spinal fluid (aCSF; composition [in mM]: 124 NaCl, 3 KCl, 1.3 MgCl_2_, 2.6 CaCl_2_, 1.25 NaH_2_PO_4_, 26 NaHCO_3_ and 10 d-glucose). Hippocampal slices (350 µM) were cut with a Leica vibrotome (VT1200S) (Richmond Hill, On, Canada) and were left at room temperature to recover for at least 1 h before being transferred to a submersion recording chamber. Field post-synaptic potential (fPSP) recordings were made from the *stratum radiatum* region of CA1 of the hippocampus using electrodes filled with aCSF (resistance 3–5 MΩ). Baseline stimulation along the Schaffer collateral pathway was accomplished with a bipolar tungsten electrode (Rhodes Medical Instruments) at a frequency of 0.05 Hz. The baseline period consisted of at least 10 min of stable recordings taken at half-maximum response strength (amplitude ≥0.5 mV). If a drug solution was applied, a period of 15 min was permitted for the drug to fully perfuse the slice before initiation of recording. After the baseline period, long-term potentiation (LTP) was induced with a stimulation protocol consisting of 10 trains of four stimuli delivered at 100 Hz every 40 ms [14]. Post-stimulation recordings were obtained for a period of 1 h. For analysis, responses were expressed as a percentage of the mean baseline fPSP slope (and hence are termed normalized fPSPs) averaged into 1-min bins.

### Source of chemicals

All chemicals were acquired from Sigma or from Tocris Bioscience, except psalmotoxin 1 (PcTx1, from BioTrend).

### Data analysis

Data were analyzed with Clampfit version 9.2 software (Axon Instruments). For whole-cell recordings, the desensitization and deactivation curves of the ASICs currents were fitted with a mono-exponential function. The amplitude of ASIC currents from most measuring sessions was normalized to the control current before application of the drug. Statistical analysis was based on unpaired or paired *t*-tests, as appropriate, or one-way analysis of variance (ANOVA). For field recordings in hippocampal slices, normalized fPSP values were averaged across the last 5 min for each slice in a group. These values were entered into the analytical software as individual cases. Primary statistical analysis was based on one-way ANOVA (p = .05). *Post hoc* analysis was performed with Tukey's Honestly Significant Difference test (p<0.05). Values are expressed as mean ± standard error of the mean (SEM).

## Results

### Activation of GABA_A_ receptors inhibits ASIC currents in hippocampal neurons

We used a whole-cell voltage-clamp configuration to record ASIC currents in cultured primary hippocampal neurons in response to repeated application of a pH 5.8 solution. The peak amplitude of whole-cell ASIC currents (evoked with pH 5.8 solution) in hippocampal neurons was variable, averaging 2.2±0.4 nA (n = 25). Under our recording conditions the responses to GABA (at 100 µM) were small relative to ASICs currents (steady-state current: 72±6 pA, n = 18) due to the small driving force on chloride at −60 mV (Figure S1).

**Figure 1:**
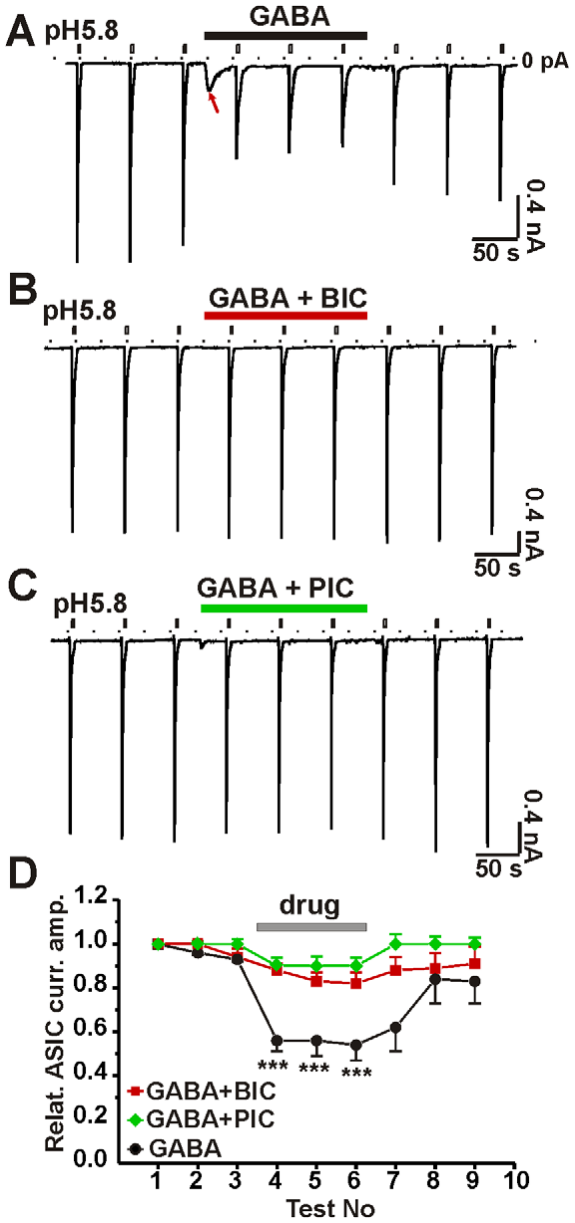
Activation of GABA_A_ receptors reversibly inhibits ASIC currents. A, ASICs were activated by pH 5.8 solution repetitively in every minute. GABA (100 µM) reversibly attenuated ASIC currents. Red arrow indicates the current activated by GABA. Persistent application of GABA desensitized a large portion of GABA_A_ receptors and steady-state GABA-current was small compared to ASIC currents (Figure S1). Dashed line indicates a position of zero current. B and C, co-application of bicuculline (BIC, 100 µM, B) or of picrotoxin (PIC, 100 µM, C) with GABA blocked GABA-activated current and abolished the inhibition of ASICs. D, statistic graph shows relative ASIC currents that were affected by GABA but reversed by antagonists of GABA_A_ receptors. n = 6–8, ***, p<0.001, T-test, GABA plus GABA antagonists vs. GABA alone.

Application of GABA reversibly inhibited ASIC currents (Figure 1), but this inhibition was eliminated when GABA_A_ receptors were blocked by application of a GABA_A_ receptor antagonist (either bicuculline or picrotoxin) (Figure 1). This result suggested that activation of GABA_A_ receptors strongly regulates ASIC currents. The general anesthetics propofol and etomidate, which are partial agonists of GABA_A_ receptors, also had similar effects on the ASICs currents. In contrast, activation of GABA_B_ receptors by application of the agonist baclofen did not inhibit these currents (Figure S2).

We initially noted that inhibition of ASICs by GABA appeared to depend on the extent of desensitization of the GABA_A_ receptors. Specifically, inhibition was less when the GABA_A_ receptors were extensively desensitized. Indeed, higher concentrations of GABA did not guarantee greater inhibition of ASICs, as the high concentrations of GABA promoted desensitization. Maximal inhibition of ASICs was caused by 30 µM GABA, a situation in which steady-state currents were relatively pronounced (Figure S3A). This result suggests that opening of the GABA_A_ receptor, rather than binding of the agonist, is required for inhibition of ASICs.

To determine if closing of the GABA_A_ receptors is essential, we activated ASICs and GABA_A_ receptors at different times, to avoid overlapping changes in conductance. When the two types of receptors were not concurrently activated, GABA_A_ receptors did not affect ASICs (Figure S3B). This result indicates that closed GABA_A_ receptors do not mediate the inhibition of ASICs.

### Intracellular chloride ions do not inhibit ASICs

**Figure 2:**
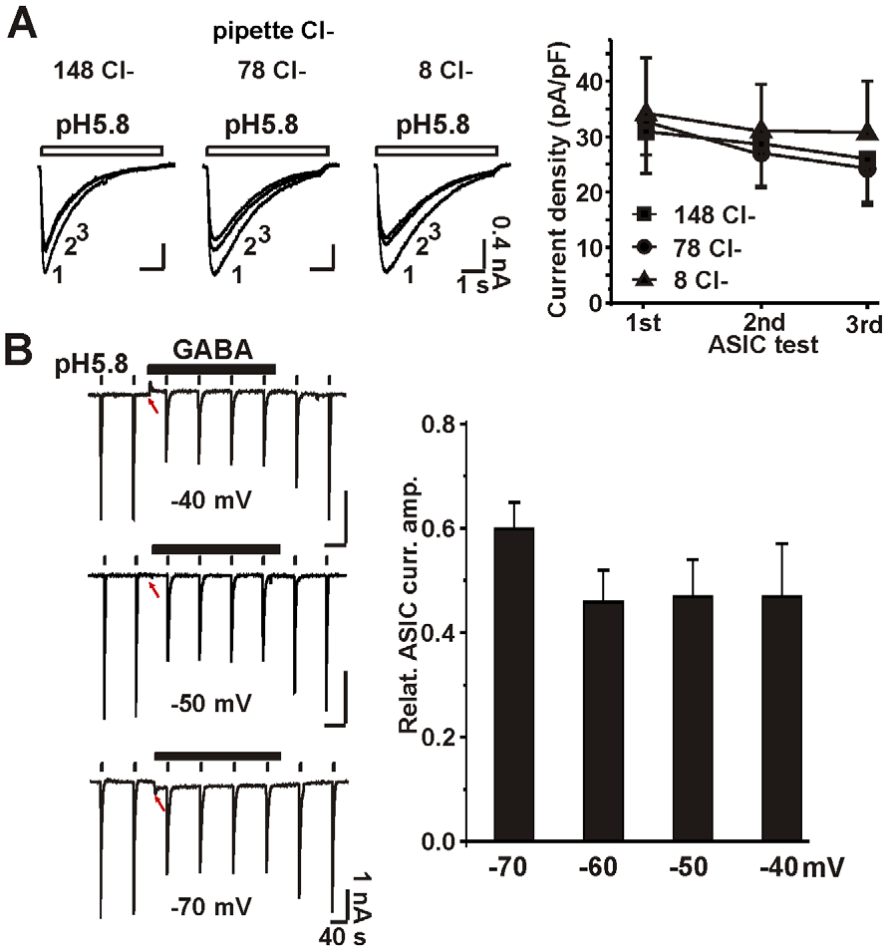
ASICs are not inhibited by intracellular chloride ions. A, Intracellular pipette solution containing different concentrations of chloride ions. The major salt components of the intracellular pipette solution were 140 mM KCl, 70 mM KCl/70 mM K-gluconate or 140 mM K-gluconate, resulting in chloride concentrations of 148, 78 and 8 mM, respectively (see Methods section for other ingredients). ASIC currents were tested three times (once per minute) immediately after the whole-cell recording configuration was established. The figure shows representative traces (left, super-imposed) and current density of ASICs (right) for the first three tests. There were no differences in the ASIC current density recorded using the three different intracellular pipette solutions. n = 11–17. ANOVA test, p = 0.95, 0.92 and 0.84 for the 1st, 2nd and 3rd, respectively. B, both direction of Cl- flux inhibit ASIC currents. Membrane potentials were clamped at −40 mV, −50 mV, −60 mV and −70 mV, respectively. The intracellular pipette solution contained 140 mM cesium gluconate (having 8 mM Cl- in recording pipettes). Left, representative traces of ASIC currents. Arrows indicate currents evoked by GABA. Note that at −50 mV, GABA elicited zero current (pointed by red arrows) but ASIC currents were still inhibited. Right, bar graph shows the ASIC inhibition by GABA at various clamping potentials. No significant differences (p = 0.34) were detected by one–way ANOVA test.

Activation of GABA_A_ receptors causes either influx or efflux of chloride ions, depending on the reversal potential for these anions. To examine whether the intracellular concentration of chloride ions regulates the activity of ASICs, we used three intracellular pipette solutions containing different concentrations of chloride, but detected no difference in the current density of ASICs (Figure 2A). For the first three applications of low pH solution (within 0–3 min after whole-cell configuration was established, when the intracellular pipette solution first enters the neuron), we observed no differences in the slight run-down of ASIC currents among the three concentrations of intracellular chloride (Figure 2A). These data indicate that high intracellular chloride does not inhibit ASICs.

To test whether influx or efflux of chloride through the open pore of GABA_A_ receptors affects ASIC activities, we clamped the membrane potential at different levels. The reversal potential for chloride ions (or anions) was −56±2 mV (n = 8) when the intracellular pipette solution contained mainly cesium gluconate. Applications of GABA evoked outward currents at −40 mV and inward currents at −70 mV. The outward current was carried by an influx of chloride ions (resulting in an increase in intracellular chloride concentration), and the inward current was carried by an efflux of anions, including chloride ions. Both the outward and the inward GABA_A_ receptor-mediated currents inhibited ASIC currents (Figure 2B). Moreover, when we clamped the membrane potential at the reversal potential for chloride ions, activation of GABA_A_ receptors evoked no net current. Figure 2B shows that the ASIC currents were still inhibited by application of GABA. Together, these data indicate that changes in intracellular concentration of chloride are not responsible for inhibition of ASIC currents. This suggests that gating of the GABA_A_ receptors may be required for inhibition to occur.

### The kinetics of ASIC currents are modified by activation of GABA_A_ receptors

**Figure 3:**
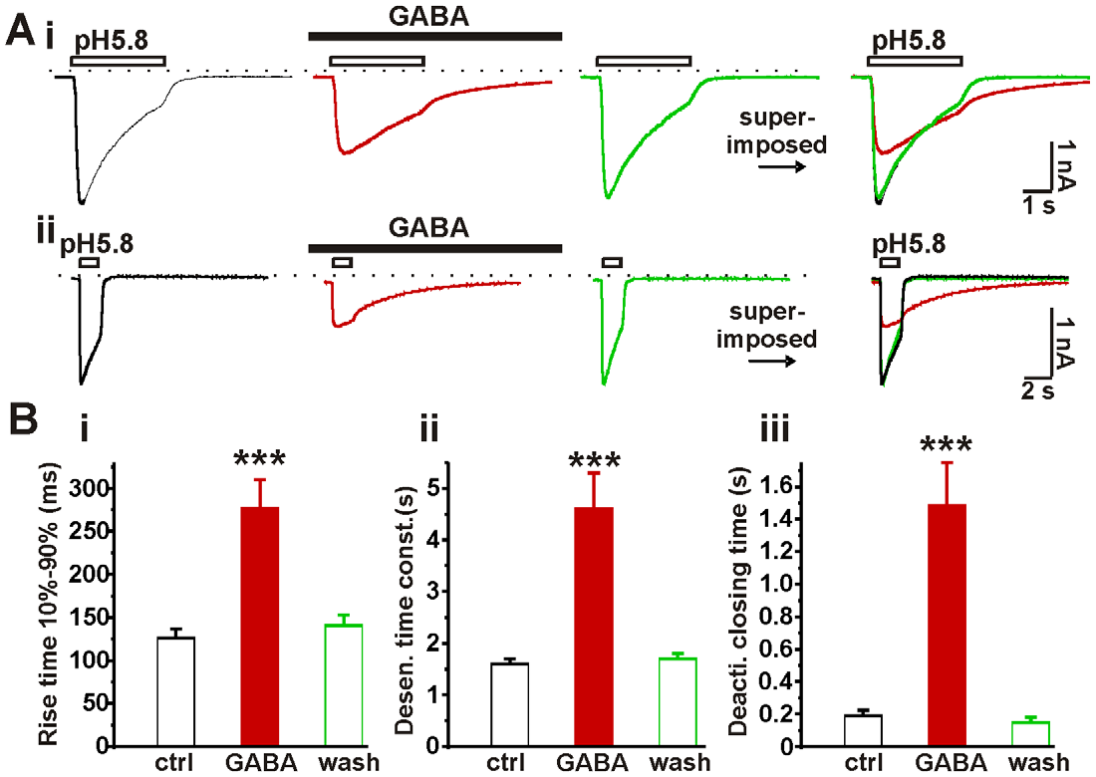
Activation of GABA_A_ receptors modifies the current kinetics of ASICs. A, two representative current traces recorded from two hippocampal neurons (i and ii), respectively. ASICs were activated with pH 5.8 for 4 seconds (i) or for shorter 1.5 seconds (ii) to monitor the deactivation process of ASIC currents, GABA (100 µM) altered the overall shape of ASIC currents. ASIC current traces were superimposed to the right without scaling. B, bar graph showing the summarized data of rise time of activation (10–90%) (i), desensitization time constant (ii) and deactivation time (iii) of ASIC currents in the absence or presence of GABA. ***, paired t-test, p<0.001, GABA group vs. control group, n = 17–20.

Activation of GABA_A_ receptors had multiple effects on the ASIC current; not only was the peak amplitude of the ASIC current attenuated, but also the kinetics of macroscopic ASIC currents were altered (Figure 3). In the absence of GABA, the rise time (10%–90%) for the ASIC currents was 126±11 ms (n = 18); conversely, in the presence of GABA, the rise time increased markedly, to 277±33 ms (p<0.001). Following washout of GABA, the rise time fell to 141±12 ms. In a second set of observations, the time constant for desensitization of ASIC currents was 1.6±0.1 s (n = 20). This value increased substantially, to 4.6±0.7 s upon activation of GABA_A_ receptors (p<0.001) and returned to 1.7±0.1 s after washout of GABA. Finally, when the pH of the solution was increased from 5.8 to 7.4 the kinetics of deactivation of the proton response was also altered. The time for deactivation from 10% to 90% was 190±34 ms (n = 17) in the absence of GABA, but it was dramatically prolonged in the presence of GABA. Deactivation in the presence of GABA could be fitted with a mono-exponential function, which yielded a time constant of 1486±263 ms. After washout of GABA, the time for deactivation (10%–90%) recovered to 150±31 ms. These data indicate that the functions of ASICs in neurons are modified by activation of GABA_A_ receptors.

### GABA modifies ASIC currents in nucleated patches and outside-out patches

**Figure 4:**
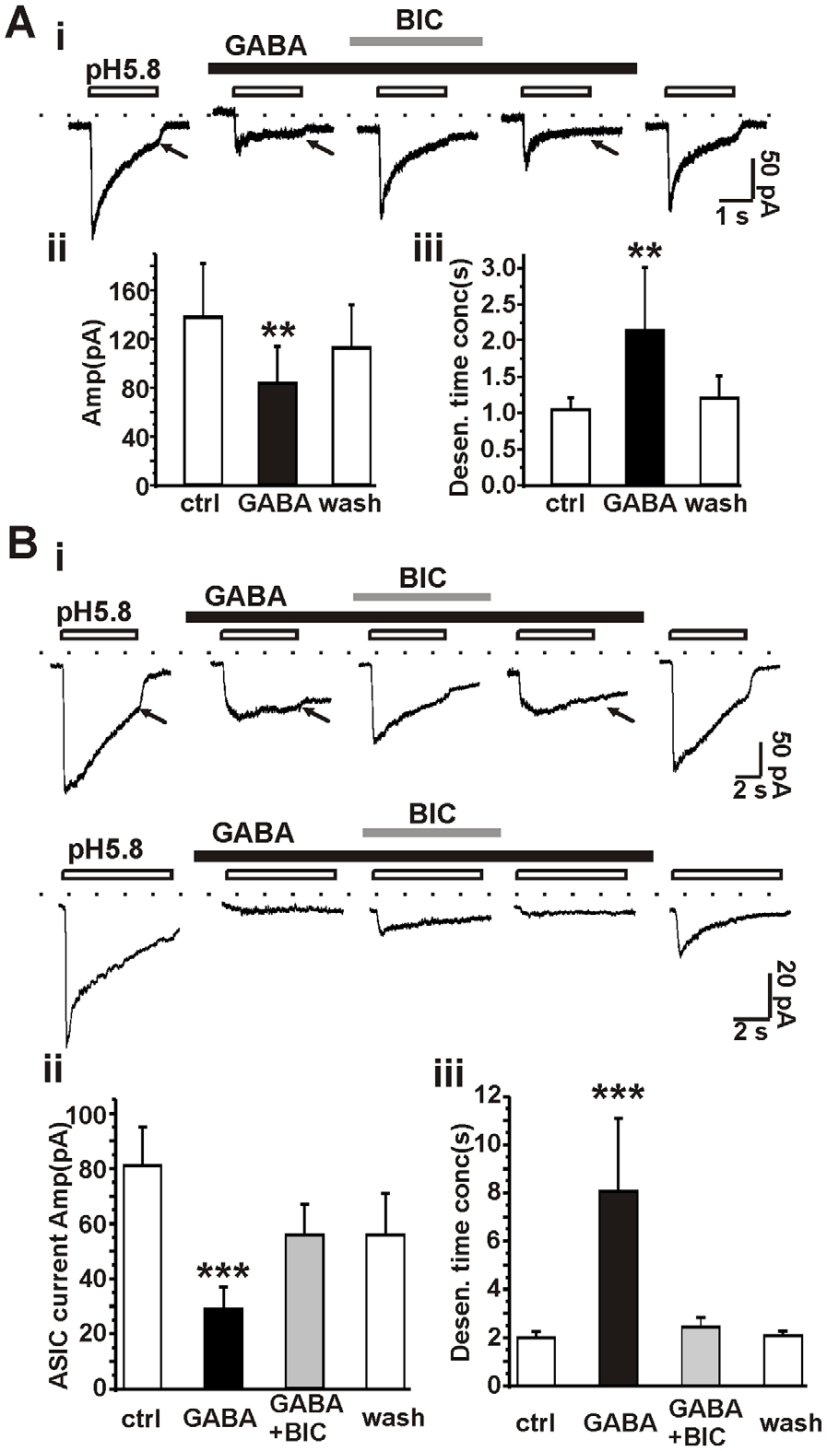
ASIC currents are modified by GABA in nucleated patches and outside-out patches excised from hippocampal neurons. A, ASIC currents recorded from nucleated patches. The peak ASIC current amplitude was −279±92 pA (n = 16). i, representative traces of ASIC current recorded from nucleated patches. GABA (100 µM) attenuated ASIC currents and rendered ASIC currents desensitized slower and inactivated incompletely (denoted by arrows). Bar graph shows the statistics of peak current amplitude (ii) and desensitization time constant (iii) of ASIC currents in the absence or presence of GABA. n = 9, **, p<0.01, GABA group versus control group. B, ASIC currents recorded from outside-out patches. The peak ASIC current amplitude were −58±7 pA (n = 36). i, two representative traces of ASIC currents recorded from outside-out patches. GABA (100 µM) attenuated the peak current amplitude and altered the current kinetics of ASICs. Arrows denote that the deactivation of ASIC currents was prolonged. Bar graph showing the summarized data of peak current amplitude (ii) and desensitization time constant (iii) of ASIC currents in the absence or presence of GABA. Blocking GABA_A_ receptors with bicuculline (100 µM) reversed the impact of GABA on ASICs. n = 14, ***, p<0.001, GABA group versus control group.

Activation of GABA_A_ receptors could decrease input resistance, which might indirectly attenuate the current amplitude of ASICs in whole-cell recordings. To improve both the space clamping of neurons and the control of concentrations of applied drugs and ions (e.g., concentration-clamp), we next performed recordings in nucleated patches excised from cultured primary hippocampal neurons. We excised 16 nucleated patches, all of which demonstrated low pH-induced ASIC responses. The ASIC currents in 7 of the 16 patches were not affected by GABA (Figure S4A), even though a GABA-related conductance was observed. This result suggests that not all ASICs are affected by GABA or that the effects of GABA may depend on specific neuronal subtypes. The peak amplitudes and the kinetics of ASIC currents in the other 9 patches were strongly modified by GABA (Figure 4A). The GABA_A_ receptor antagonist bicuculline abolished these effects of GABA on ASIC currents. The peak amplitude of ASIC currents was 133±44 pA (n = 9) before application of GABA and was reduced to 80±30 pA (p<0.01) in the presence of GABA, recovering to 113±35 pA after washout of GABA. GABA also increased the time of ASIC desensitization. The desensitization time constants were 1.05±0.16 s, 2.14±0.87 s and 1.21±0.30 s (n = 4) before, during and after GABA application, respectively.

To further test if the modification of ASICs by GABA occurred in a cell-free condition, we next performed voltage-clamp recordings in outside-out patches excised from hippocampal neurons. We obtained a total of 36 ASIC-positive outside-out patches. We tested the effect of GABA in 18 of the patches and the effect of muscimol, a selective GABA_A_ receptor agonist, in the other 18 patches. GABA modified the ASIC currents in 14 out of 18 patches. The current amplitudes of ASICs were markedly attenuated, declining from 81±14 pA to 29±8 pA (n = 14) (p<0.001) (Figure 4B). Moreover, the desensitization time constant of the ASIC currents increased substantially, from 2.01±0.22 s to 8.03±3.02 s (p<0.001). Both of these effects of GABA were reversed by bicuculline. Application of GABA had no detectable effect on ASIC currents in the other 4 patches, although both ASIC- and GABA-related responses were detected (Figure S4B), the latter suggests that the interaction of ASICs and GABA_A_ receptors may require interactions with specific microdomains. Muscimol had similar regulatory affects on ASIC currents in 8 of the 18 outside-out patches, but no effect in the other 10 patches (Figure S5). About half of all outside-out patches demonstrated a pH-activated ASIC response. Together, these data confirm that activation of GABA_A_ receptors leads to modification of ASICs currents, even in excised patches.

### Activation of glycine receptors in spinal neurons modifies ASIC currents

**Figure 5:**
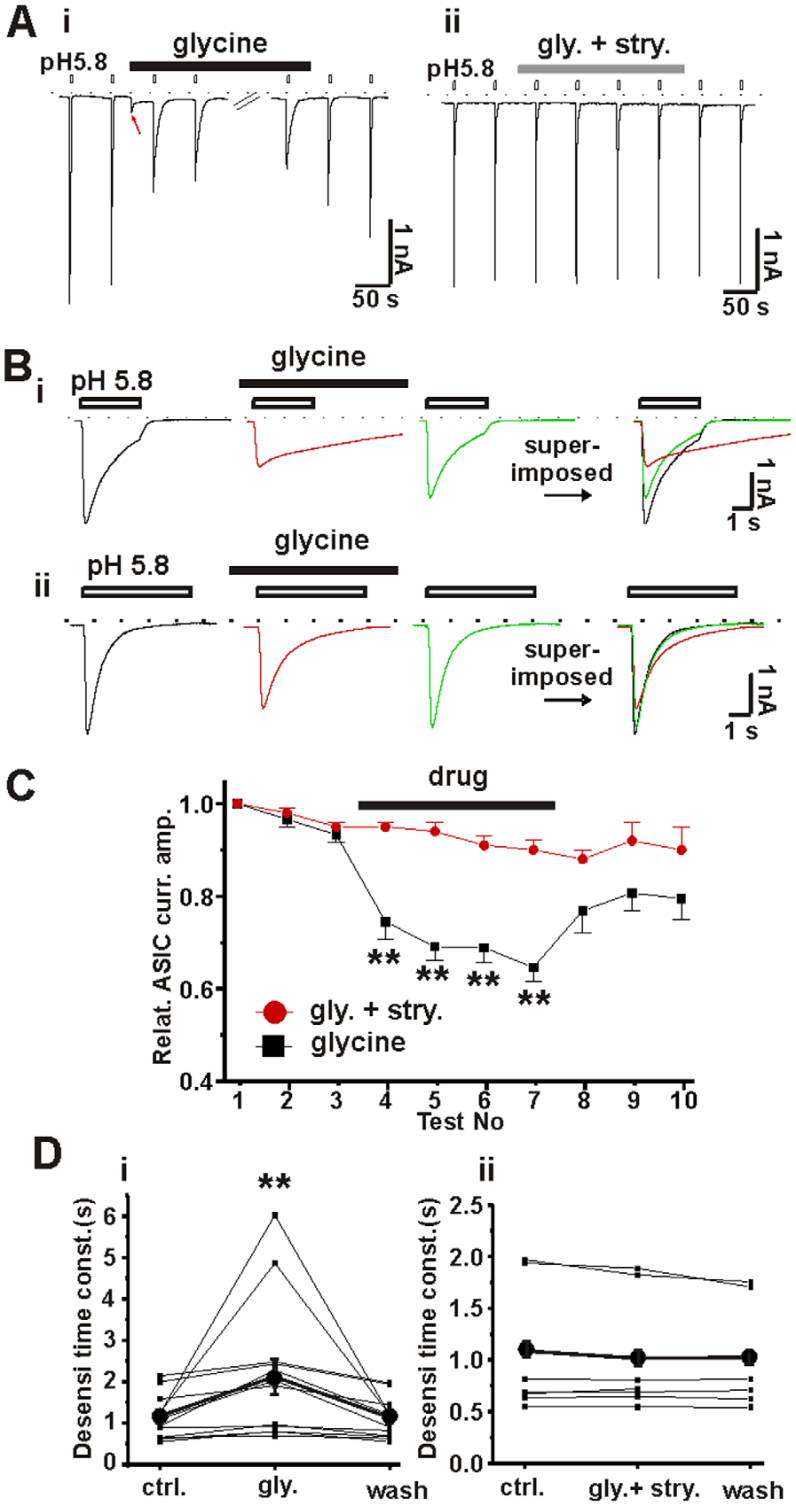
Activation of glycine receptors modifies ASICs in spinal neurons. A, representative traces of ASIC currents (elicited by pH 5.8) recorded in cultured primary spinal neurons. Application of glycine (500 µM) reversibly attenuated ASIC currents (i) and strychnine (10 µM) abolished the effect of glycine on ASICs (ii). Red arrow indicates the glycine-evoked current, which desensitized and remained small in the presence of glycine. Dashed lines indicate the position of zero current. B, two representative current traces (i & ii from different spinal neurons) showing the variable effect of glycine. Glycine had dramatic effect on desensitization kinetics and current amplitude of ASICs in some spinal neurons (example i) but had weak effect in other neurons (example ii). The right parts were superimposed traces without scaling. C, statistic data shows relative ASIC current amplitude that was affected by glycine application but was reversed by strychnine. n = 5–8, **, p<0.01, unpaired t-test. D, (i) the desensitization time constant of ASIC currents was increased by activation of glycine receptors. n = 12, **, p<0.01, paired t-test; glycine group vs. control group. (ii) strychnine abolished the effect of glycine on ASIC desensitization. n = 12, p = 0.35, pared t-test. The mean values were shown in filled circles.

Glycine receptors are ligand-gated chloride channels that are abundantly expressed in spinal neurons [15]. To determine if activation of glycine receptors modifies ASICs, we recorded ASIC currents from cultured primary spinal neurons. The degree of inhibition of ASIC by applications of glycine was variable, but some inhibition was observed in most recordings (Figure 5A&B). Strychnine, an antagonist of glycine receptors, abolished glycine-induced ASIC inhibition. Furthermore, activation of glycine receptors markedly increased the desensitization time constant of ASIC currents from 1.3±0.2 s to 2.2±0.5 s. This effect could be abolished by strychnine (Figure 5A&C). Similarly, the deactivation kinetics of ASIC currents was also slowed by glycine (Figure 5D). These data indicate that ASICs can be modified by activation of another ligand-gated chloride channel.

### Activation of GABA_A_ receptors alters the pharmacology of ASIC currents

**Figure 6:**
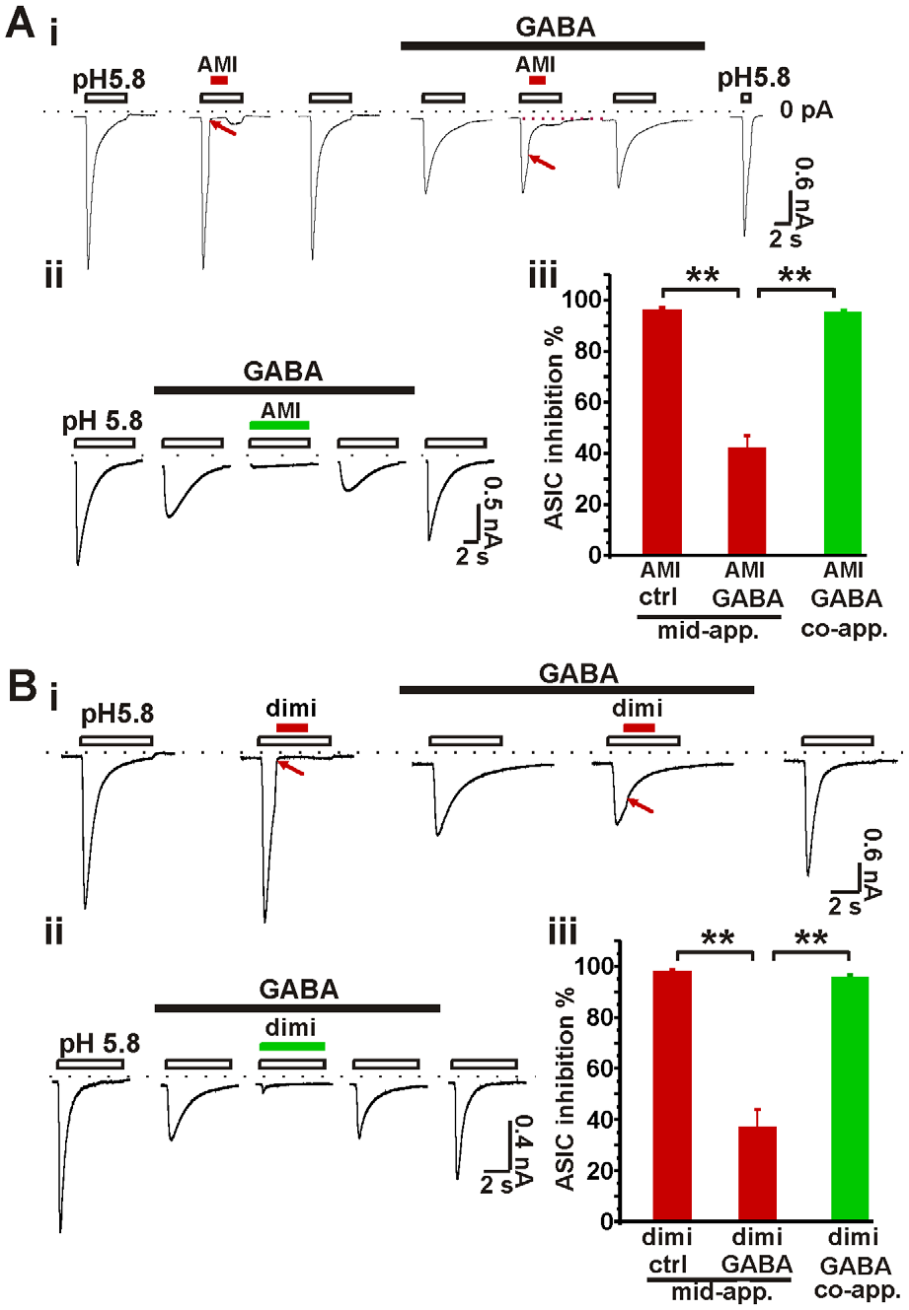
The pharmacology of ASICs is modified by GABA. A, the ASIC blockade by amiloride was altered by activation of GABA_A_ receptors. i, representative traces of ASIC currents. Middle-applied amiloride (AMI, 200 µM) fully blocked ASIC currents. Application of GABA (100 µM) attenuated the ASIC currents and also reduced the degree of blockade by middle-applied amiloride, n = 8. Red arrows denote the difference of ASIC blockade by amiloride in the absence or presence of GABA. ii, in the presence of GABA, co-applied amiloride (AMI, 200 µM) fully blocked ASIC currents, n = 7. iii, bar graph showing a summary of the blockade of ASICs by amiloride (middle-applied vs. co-applied) in the absence or presence of GABA. **, p<0.01. B, the ASIC blockade by diminazene was altered by activation of GABA_A_ receptors. i, representative traces of ASIC currents. Middle-applied diminazene (dimi, 20 µM) completely blocked ASIC currents. Application of GABA (100 µM) attenuated the ASIC blockade by the middle-applied diminazene, n = 6–7. ii, representative current traces showing that in the presence of GABA, co-applied diminazene (dimi, 20 µM) fully blocked ASIC currents, n = 7. iii, bar graph showing the statistics of the inhibition of ASICs by diminazene (middle-applied vs. co-applied) in the absence or presence of GABA. **, p<0.01. ASIC current at the presence of blockers was normalized to a measured ASIC current before applying blockers, which thereby obtained the inhibition percentage. mid-app. (middle-applied drug): first activated ASICs with pH 5.8, then applied blocker when part of ASICs were still open. co-app. (co-applied): ASIC blocker and pH 5.8 solution were applied simultaneously.

We next sought to determine whether the pharmacological properties of ASIC currents are modified by activation of GABA_A_ receptors in hippocampal neurons. We tested the blockade of ASIC currents by amiloride [2] or diminazene [16] in the absence or presence of GABA. Neither of these agents inhibited GABA_A_ receptor-mediated currents when administered on its own (Figure S6). Amiloride (200 µM, briefly applied mid-way during application low pH solution) strongly inhibited ASIC currents by 96%±1% (n = 8) (Figure 6A). However, after application of GABA, amiloride (200 µM) was less effective at inhibiting ASIC currents (inhibition by 42%±5%; n = 8). In comparison, simultaneous application of amiloride (200 µM) with GABA led to full blocking of ASICs currents. These data suggest that the pharmacological properties of ASICs are modified by activation of GABA_A_ receptors. We then used diminazene, which is structurally distinct from amiloride [16], to validate these results. Application of diminazene (20 µM, briefly applied mid-way during application of low pH solution) blocked ASIC currents by 97%±2% in the absence of GABA (Figure 6B). However, in the presence of GABA, the same compound failed to fully block the ASIC currents, and the percent inhibition was reduced to 37%±7%. When diminazene (20 µM) was applied simultaneously with GABA, ASIC currents were blocked (inhibition 95%±1%). These results argue against the possibility that protons *per se* potentiate GABA_A_ receptors. These data reveal that the pharmacological features of ASICs are modified when GABA_A_ receptors are gated.

### ASICs regulate synaptic plasticity in coordination with GABA_A_ receptors

**Figure 7:**
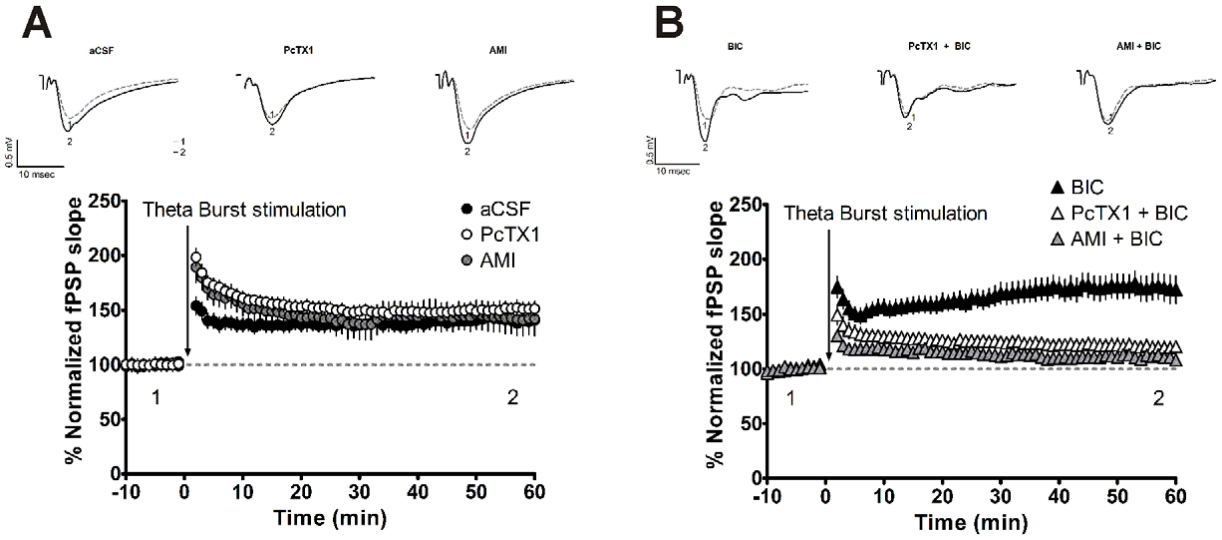
ASICs and GABA_A_ receptors coordinate to regulate synaptic plasticity in CA1. A, robust LTP was elicited using a theta burst stimulation protocol (in aCSF condition, filled circles). This LTP was not affected by ASIC inhibition with amiloride (gray circles, 100 µM) or with PcTx1 (empty circles, 30 nM). B, simultaneous inhibition of GABA_A_ receptors and ASICs impeded LTP. Blockade of GABA_A_ with bicuculline markedly increased LTP (filled triangles). Co-application of bicuculline either with amiloride (gray triangles) or with PcTx1 (empty triangles) resulted in an attenuation of bicuculline-enhanced LTP (filled triangles). Overlays for A and B show representative responses before and after stimulation (top). Normalized slope of fPSP were shown in the bottom. Abbreviations: aCSF = artificial cerebral spinal fluid; AMI = amiloride; BIC = bicuculline; PcTx1 = psalmotoxin 1.

ASICs have been implicated in synaptic plasticity, as knocking out the ASIC1 gene impairs LTP of Schaffer-collateral CA1 synapses [3]. We performed field recordings from hippocampal slices to determine whether ASICs and GABA_A_ receptors interact to regulate LTP in a coordinated manner at CA1 Schaffer-collateral synapses. We used amiloride or the ASIC1a-specific blocker PcTx1 to inhibit ASICs and bicuculline to inhibit GABA_A_ receptors. LTP was elicited under control conditions (aCSF, 138.6%±9.1%, n = 11) by a theta burst stimulation protocol (Figure 7A). Amiloride or PcTx1 applied alone did not attenuate the LTP (amiloride: 141.9%±15.0%, n = 8; PcTx1: 151.5%±6.2%, n = 9). In contrast, blockade of GABA_A_ receptors with bicuculline enhanced the LTP to 173.9%±12.0% (n = 17) (Figure 7B). When amiloride and bicuculline were applied simultaneously, the LTP was reduced to 111.9%±4.8% (n = 12). Similarly, when PcTx1 and bicuculline were applied simultaneously, the LTP decreased to 119.7%±5.9% (n = 12). Thus, in both of these conditions, the potentiation was much less than that observed with bicuculline alone (Figure 7B). These data indicate that simultaneous blockade of both ASICs and GABA_A_ receptors suppressed the bicuculline-enhanced LTP, in turn suggesting that ASICs and GABA_A_ receptors may team up to regulate synaptic plasticity.

## Discussion

In the study reported here, activation of GABA_A_ receptors strongly modified ASIC currents in hippocampal neurons. These modifications included attenuation of peak current amplitude, slowing of kinetics and alteration of sensitivity to ASIC blockers. Similar effects on ASICs were observed with glycine receptors in spinal neurons. Furthermore, the inhibition of ASICs by GABA_A_ also attenuated LTP at CA1 synapses. Our overall conclusion is that ASICs in CNS neurons are modified by open ligand-gated chloride channels.

### GABA_A_ receptors modify ASICs, most likely though conformation-dependent interaction

The question arises as to how the gating of GABA_A_ receptors modifies ASICs in CNS neurons. Modification of ASICs by activation of GABA_A_ receptors occurred rapidly, and when the chloride channels were closed or became desensitized, the modifications were eliminated or reduced, respectively. The ASIC currents also recovered rapidly (Figure 1). In addition, regulation of ASICs by GABA was observed in outside-out patches. GABA_B_ receptors did not mediate the inhibition of ASICs. Thus, it is unlikely that the modification of ASICs by GABA is mediated by GPCR-mediated intracellular signaling messengers.

Our data also argue against the possibility that modification of ASICs is caused simply by an increase in intracellular chloride, because both outward and inward GABA-induced current inhibited the ASIC currents. Moreover, when activation of GABA_A_ receptors effectively conducted no net flux of chloride ions, the ASIC currents were still modified by conducting GABA_A_ receptors (Figure 2B). In addition, we detected no differences in ASIC current density as a consequence of manipulating intracellular chloride concentration. Hence, we can conclude that simply a change in intracellular chloride is not responsible for the ASIC modifications. Nevertheless, we cannot rule out a possibility that extracellular chloride or the concentration gradient of chloride cross the plasma membrane exert some effect on ASICs when GABA_A_Rs stay open.

This study suggests a model of conformation-dependent interaction between GABA_A_ receptors and ASICs (Figure S7). That is, upon binding of GABA, the GABA_A_ receptors undergo a conformational change. Binding of protons converts the conformation of ASICs to the open state and at this point the ASICs may interact with the open GABA_A_ receptors. The interaction of two open receptors results in depression of the typical ASIC currents. Furthermore, there are modifications of the kinetics of activation, desensitization and deactivation and even the pharmacology of ASICs. Activation of GABA_A_ receptors prevents blockade (by amiloride or diminazene) of about 60% of the attenuated ASIC currents (Figure 6), an effect that may be caused by interaction of open ASICs with open GABA_A_ receptors. The emergence of ASIC-blocker-insensitive current probably accounts for the overall change in the kinetics of ASIC currents (Figure 3). This tentative model requires that GABA_A_ receptors and ASICs be located in close proximity to enable their interaction. Recordings from nucleated and outside-out patches provided evidence supporting this model (Figure 4).

We do not argue that ASICs must directly interact with GABA_A_Rs. Although plausibly these two receptors may physically couple that may depend on their conformation change, an alternative explanation is that an intermediate protein or regulator might participate in this receptor-receptor interaction. This intermediate protein may be affected by the conformation change of GABA_A_Rs which in turn influences ASICs. We have not identified this putative protein, thus more experiments are needed for a clear mechanistic scenario.

### Comparison of the ASIC regulation by GABA_A_ receptors with the ENaC regulation by CFTR

ASICs and ENaC have substantial homology, and both belong to the ENaC/DEG gene family [2] [17]. In this section, we compare the interaction of ASICs with GABA_A_ receptors, as elucidated in the studies reported here, with the interaction of ENaC with CFTR. Firstly, in both of these interactions, chloride channels (GABA_A_/glycine receptors or CFTR) regulate two voltage-independent sodium channels (ASICs or ENaC, respectively). Secondly, chloride has been found to bind to the conserved cysteine-rich domain of ASIC1 [7], [8], which probably implies a role of extracellular chloride in this form of regulation. Similarly, activation of CFTR is required for the regulation of ENaC [18]. Thirdly, CFTR has recently been found to impede the protease cleavage process of ENaC, which suggests that CFTR modifies ENaC [19], similar to the modification of ASICs by GABA_A_ receptors. Fourthly, various mechanistic models, typically models of direct association of CFTR with ENaC, have been proposed and substantiated [20]. Fifthly, CFTR influences ENaC in various ways and can even promote ENaC activity in sweat glands [21]. Similarly, activation of GABA_A_ receptors exerts multifaceted effects on ASICs (Figure 4B and Figure S4). Taken together, it appears that the interaction of ASICs with GABA_A_ receptors in neurons resembles in some ways the interaction of ENaC and CFTR in epithelia.

### Physiological and pathophysiological implications of the interaction between ASICs and GABA_A_ receptors

Under persistent exposure to moderately low pH (e.g., 6.9), most ASICs in CNS neurons undergo steady-state desensitization and remain in an inactive conformation [22] [23]. This feature makes it difficult to explain some of the pathological roles of ASICs, such as ischemic neuronal death [5]. The concentration of GABA increases drastically during focal ischemia and remains elevated for 2–4 h after reperfusion [24]. After stroke in mice, tonic neuronal inhibition is increased in the peri-infarct zone, and reducing excessive GABA-mediated tonic inhibition promotes functional recovery after stroke [25]. Here, we have demonstrated that ASICs interact with open GABA_A_ receptors, with modification of their biophysical features. Importantly, the times for desensitization and deactivation were dramatically prolonged by persistent exposure of GABA, which suggests that ASIC currents in some (if not all) neurons are more sustainable in the presence of GABA. These results also imply that the contribution of ASICs to excitotoxicity or to ischemic neuronal death could be different if the effect by GABA is taken into account.

We have also shown that blockade of ASICs does not affect LTP at Schaffer collateral-CA1 pyramidal cell synapses. However, when the activity of GABA_A_ receptors was abolished and LTP was thereby enhanced [26], the effect of ASICs on LTP emerged and ASIC blockade could then strongly attenuate LTP. This findings suggest that ASICs could serve in regulating synaptic plasticity [3], probably through interaction with GABA_A_ receptors. However, the exact mechanisms of involvement of ASICs in LTP are not well understood. Regardless, ASICs are abundantly and widely expressed in many regions, including the spinal cord, the hippocampus, the cortex, the amygdala and the cerebrum [2] [27]. Moreover, ASICs have a higher expression in GABAergic interneurons than in the principal neurons [6] [28], and ASICs play some role in promoting termination of seizures [6]. Given that the GABA_A_ receptors are the predominant inhibitory ionotropic receptors in the CNS, the interaction between ASICs and GABA_A_ receptors may occur at numerous locations and could be involved in a number of brain functions. Furthermore, it has not been established whether protons in the synaptic vesicles provide endogenous ligands for the ASICs. Several studies have focused on the expression and influence of ASICs at glutamatergic synapses [3], [29] [30]. The results of the current study, however, suggest that ASICs function in the inhibitory synapses. More specifically, a portion of ASICs could interact with GABA_A_ receptors, and ASICs might have profound impacts on GABAergic synaptic transmission that warrant further investigation.

## Supporting Information

Figure S1 Steady-state GABA_A_ receptors-mediated current is very small. Long period application (>1 min) of GABA (100 µM) only sustained a small current which were revealed by bicuculline blockade (BIC, top) or by brief GABA-washout (bottom). ASIC currents are shown as a comparison.
(TIF)

Figure S2 A-type GABA receptors mediate the modulation of ASICs. Baclofen (40 µM) did not affect ASIC currents. But etomidate (50 µM) or propofol (1 mM) reversibly inhibited ASIC currents. Bar graph shows relative peak current amplitude of ASICs that were affected by agonists of GABA_B_ or of GABA_A_ receptors. **, p<0.01; ***, p<0.001, unpaired *t*-test (drug group versus control group). The relative current amplitudes of ASICs were 0.88±0.01, n = 6 (control); 0.93±0.03, n = 8 (baclofen); 0.76±0.04, n = 8 (etomidate); and 0.49±0.04, n = 6 (propofol), respectively.
(TIF)

Figure S3 The opening of GABA_A_ receptors is critical for the ASIC inhibition. A, the concentration-response of GABA in inhibiting ASIC currents. Left, two representative traces showing that application of GABA (30 and 500 µM) inhibited ASIC currents. Red arrows indicate the peak response of GABA. Right, The effect of various concentrations of GABA on ASICs. n = 5–8. The current amplitude of ASICs in the presence of GABA was normalized to the amplitude of ASIC currents before GABA application. B, GABA (500 µM) and pH 5.8 were used to activate GABA_A_ receptors and ASICs differently (no overlapping activation of both). Left, representative current traces; right, relative ASIC currents and GABA-currents. n = 7.
(TIF)

Figure S4 GABA does not affect ASICs in some of recordings from nucleated patches and outside-out patches. A, nucleated patches. Representative traces of ASIC currents (i) and bar graph showing the statistics of current amplitude (ii) and desensitization time constant (iii) of ASICs in the absence or presence of GABA. Application of GABA (100 µM) did not affect the current amplitude (p = 0.36, paired *t*-test, n = 7) and desensitization time constant (p = 0.12, paired *t*-test) of ASICs in these recordings. The current amplitude of ASICs were 460±187 pA, 448±177 pA and 496±206 pA before, during and after GABA application, respectively. The desensitization time constants of ASICs were 747±320 ms, 694±320 ms and 787±399 ms before, during and after GABA application, respectively. B, outside-out patches. Representative whole traces (i) and scaled traces (ii) of ASIC currents. Arrow denotes the current activated by GABA. Bar graph showing the statistics of current amplitude (iii) and desensitization time constant (iv) of ASICs in the absence or presence of GABA. Application of GABA (100 µM) did not affect the current amplitude (p = 0.22, paired *t*-test) and desensitization time constant of ASICs (p = 0.96, paired *t*-test) in these outside-out patches (n = 4). The current amplitude of ASICs were 61±19 pA, 65±21 pA and 67±22 pA before, during and after GABA application, respectively. The desensitization time constants of ASICs were 972±120 ms, 962±62 ms and 897±43 ms before, during and after GABA application, respectively.
(TIF)

Figure S5 Muscimol modulates ASICs in some recordings (A) but not in other recordings (B) in outside-out patches. A, left, representative traces of ASIC currents in the absence or presence of muscimol; right, bar graph showing the peak amplitude of ASIC currents with or without muscimol. In these outside-out patch recordings (n = 8), muscimol markedly (p<0.01, paired *t*-test) decreased the current amplitude of ASICs from 37±10 pA to 12±5 pA, which were recovered to 33±9 pA after washout of muscimol. B, left, representative traces of ASIC currents in the absence or presence of muscimol; right, bar graph showing the peak amplitude of ASIC currents. In these recordings, muscimol did not (p = 0.44, paired *t*-test) affect the current amplitude of ASICs (n = 10). The current amplitude of ASICs were 44±5 pA, 43±6 pA and 47±6 pA before, during and after muscimol application, respectively.
(TIF)

Figure S6 Amiloride and diminazene do not block GABA_A_ receptors. A left, representative traces of GABA-evoked currents (100 µM) in the absence (black) or presence (red) of amiloride (AMI, 200 µM). Right, bar graph showing the relative GABA-current in the absence or presence of amiloride. Amiloride did not (p = 0.22, paired *t*-test; n = 4) affect GABA_A_ receptors. The relative peak amplitudes of GABA-currents were 0.91±0.04 and 0.91±0.02 during amiloride application and after washout of amiloride, respectively. B left, representative traces of GABA-currents in the absence (black) or presence (red) of diminazene (dimi, 50 µM). Right, bar graph showing the relative GABA-current with or without diminazene. p = 0.63, paired *t*-test, n = 5. The relative peak amplitudes of GABA-currents were 1.07±0.10 and 1.06±0.11 during diminazene application and after washout of diminazene, respectively.
(TIF)

Figure S7 A working model of interaction of ASICs with GABA_A_ receptors. In the resting states (top), GABA_A_ receptors do not intervene with the function of ASICs. When GABA_A_ receptors are opened (bottom), part of receptors that locate close to ASICs may interact with the open ASICs and thereby modify ASIC functions. This interaction may be conformation-dependent. It is unknown whether the chloride ions (red circles) bound in the extracellular thumb domains of ASICs participate in the interaction. GABA_A_ receptors may not affect ASICs if two receptors locate distantly.
(TIF)
