# Supplementary figures and images for: Functional Modifications of Acid-Sensing Ion Channels by Ligand-Gated Chloride Channels
Source: PLoS One. 2011 Jul 18;6(7):e21970. doi: 10.1371/journal.pone.0021970 (PMC3138761; doi:10.1371/journal.pone.0021970)

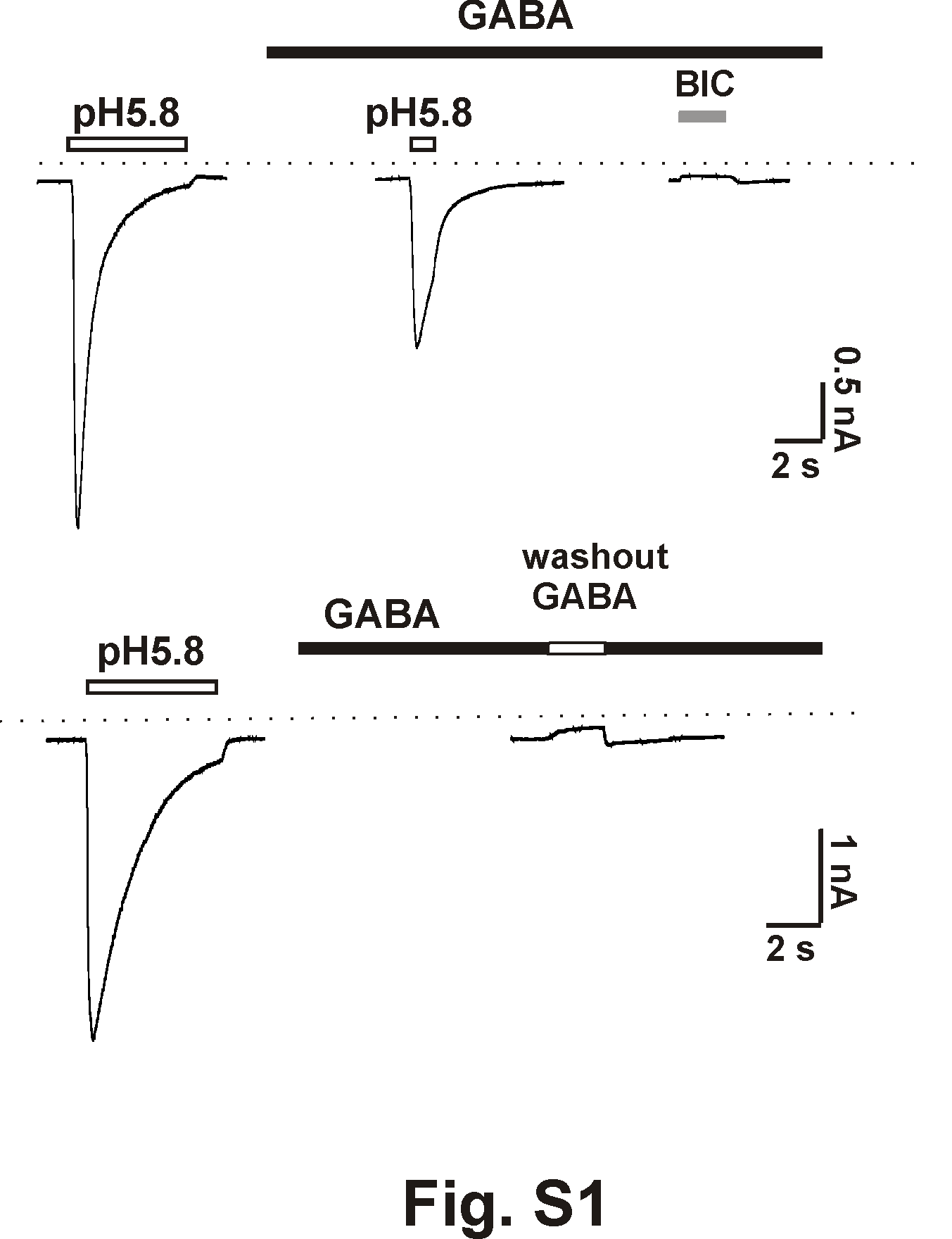

Supplement: Figure S1 — Steady-state GABAA receptors-mediated current is very small. Long period application (>1 min) of GABA (100 µM) only sustained a small current which were revealed by bicuculline blockade (BIC, top) or by brief GABA-washout (bottom). ASIC currents are shown as a comparison. (TIF) [file pone.0021970.s001.tif]

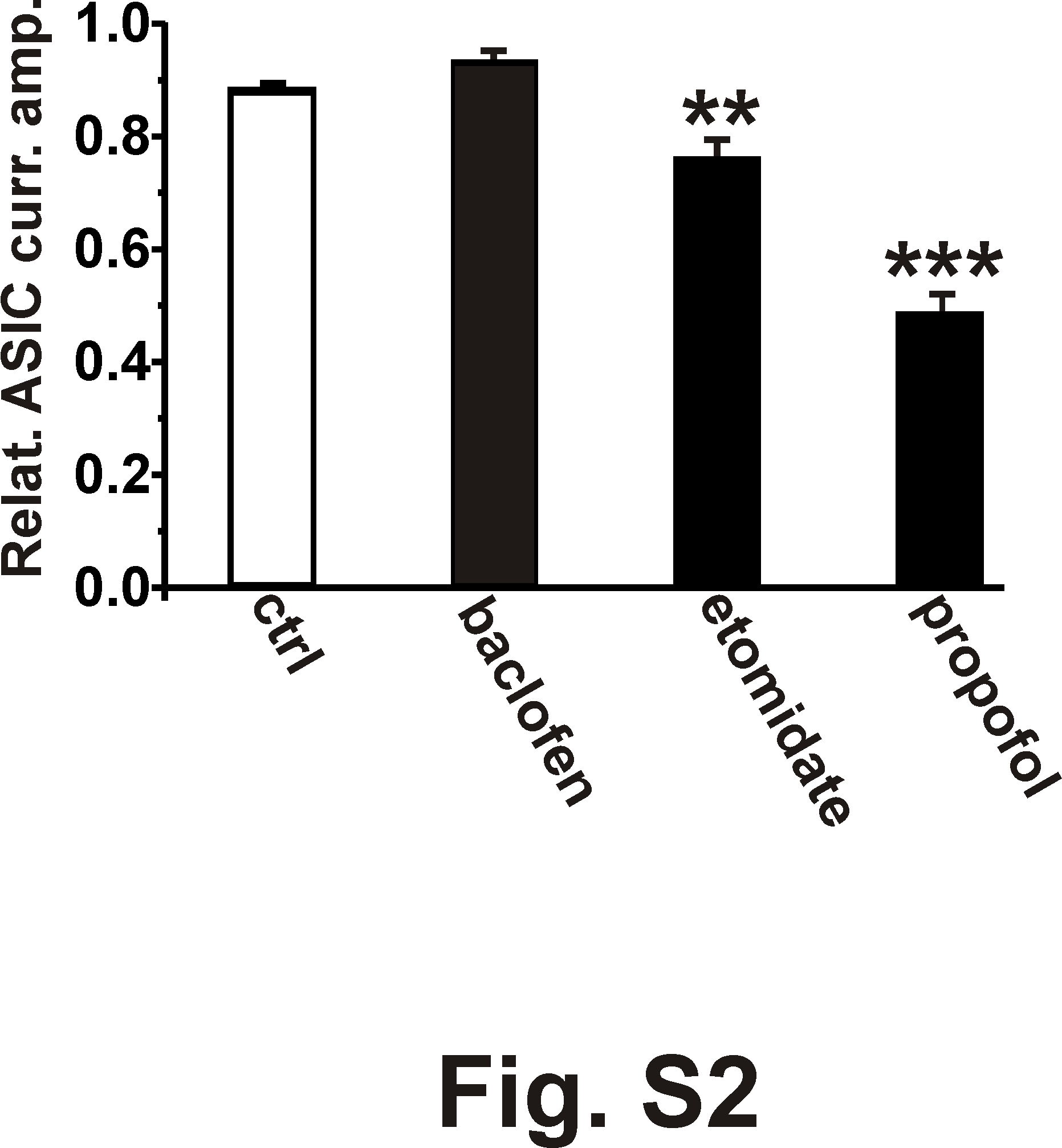

Supplement: Figure S2 — A-type GABA receptors mediate the modulation of ASICs. Baclofen (40 µM) did not affect ASIC currents. But etomidate (50 µM) or propofol (1 mM) reversibly inhibited ASIC currents. Bar graph shows relative peak current amplitude of ASICs that were affected by agonists of GABAB or of GABAA receptors. **, p<0.01; ***, p<0.001, unpaired t-test (drug group versus control group). The relative current amplitudes of ASICs were 0.88±0.01, n = 6 (control); 0.93±0.03, n = 8 (baclofen); 0.76±0.04, n = 8 (etomidate); and 0.49±0.04, n = 6 (propofol), respectively. (TIF) [file pone.0021970.s002.tif]

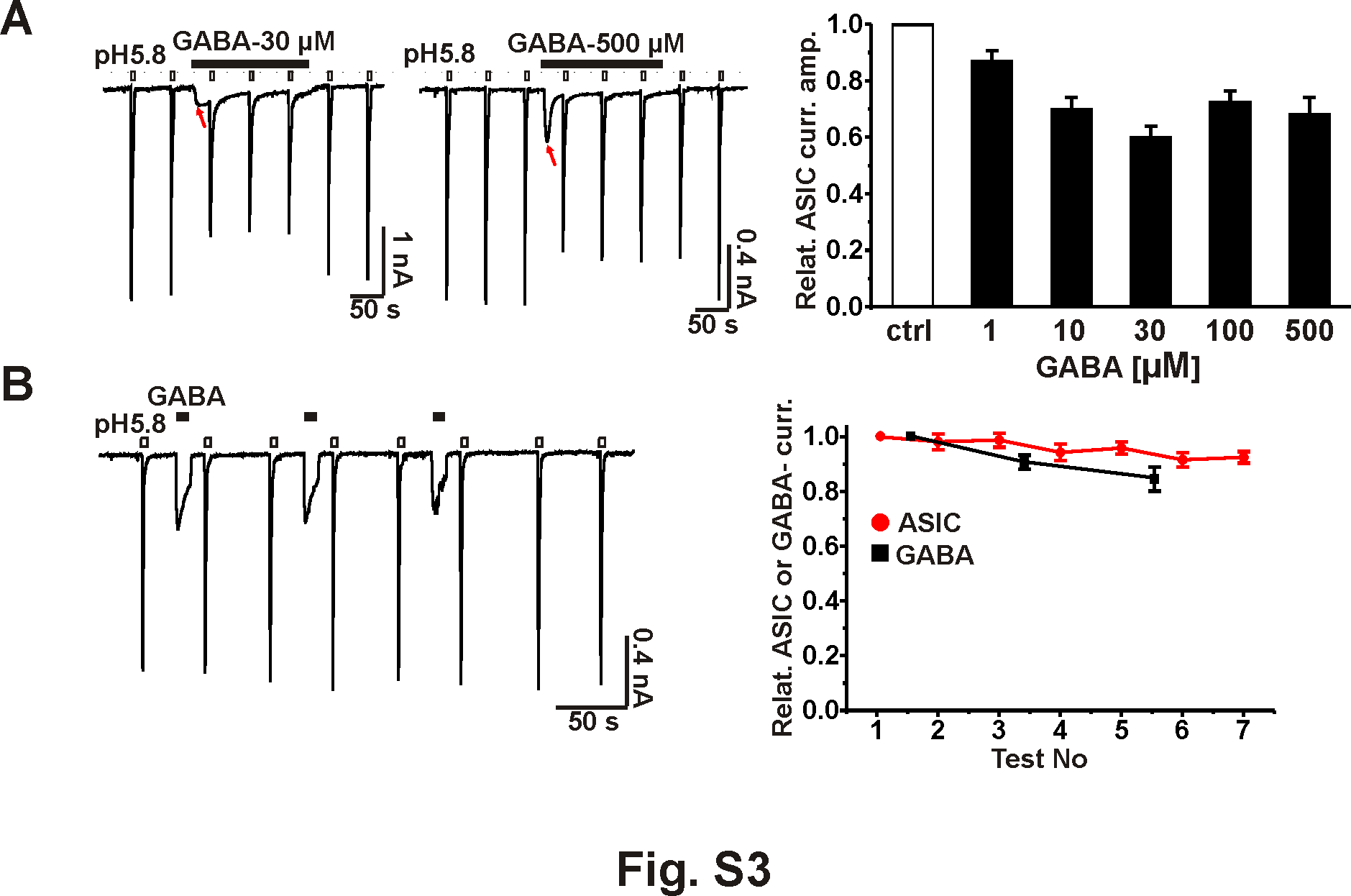

Supplement: Figure S3 — The opening of GABAA receptors is critical for the ASIC inhibition. A, the concentration-response of GABA in inhibiting ASIC currents. Left, two representative traces showing that application of GABA (30 and 500 µM) inhibited ASIC currents. Red arrows indicate the peak response of GABA. Right, The effect of various concentrations of GABA on ASICs. n = 5–8. The current amplitude of ASICs in the presence of GABA was normalized to the amplitude of ASIC currents before GABA application. B, GABA (500 µM) and pH 5.8 were used to activate GABAA receptors and ASICs differently (no overlapping activation of both). Left, representative current traces; right, relative ASIC currents and GABA-currents. n = 7. (TIF) [file pone.0021970.s003.tif]

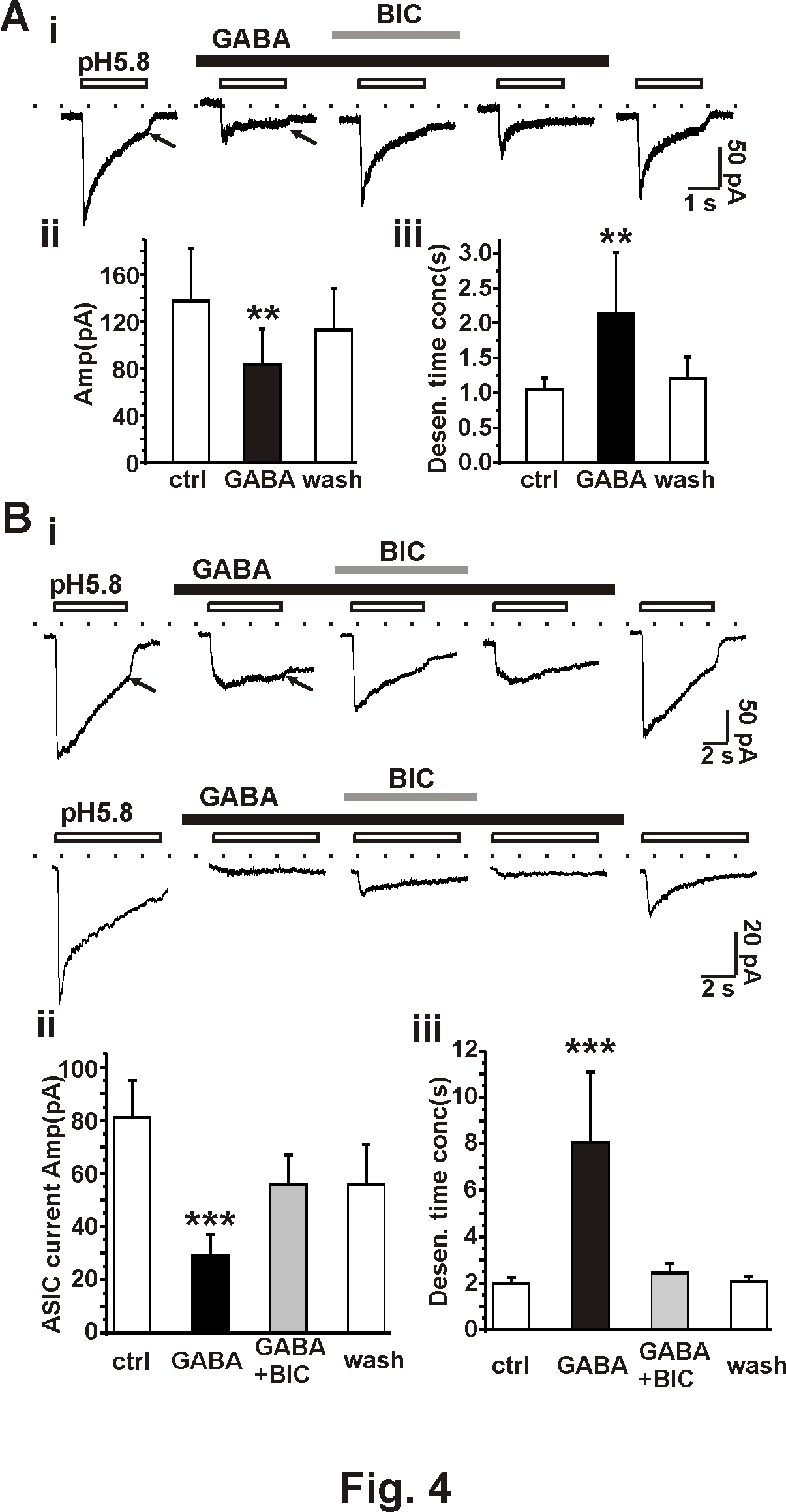

Supplement: Figure S4 — GABA does not affect ASICs in some of recordings from nucleated patches and outside-out patches. A, nucleated patches. Representative traces of ASIC currents (i) and bar graph showing the statistics of current amplitude (ii) and desensitization time constant (iii) of ASICs in the absence or presence of GABA. Application of GABA (100 µM) did not affect the current amplitude (p = 0.36, paired t-test, n = 7) and desensitization time constant (p = 0.12, paired t-test) of ASICs in these recordings. The current amplitude of ASICs were 460±187 pA, 448±177 pA and 496±206 pA before, during and after GABA application, respectively. The desensitization time constants of ASICs were 747±320 ms, 694±320 ms and 787±399 ms before, during and after GABA application, respectively. B, outside-out patches. Representative whole traces (i) and scaled traces (ii) of ASIC currents. Arrow denotes the current activated by GABA. Bar graph showing the statistics of current amplitude (iii) and desensitization time constant (iv) of ASICs in the absence or presence of GABA. Application of GABA (100 µM) did not affect the current amplitude (p = 0.22, paired t-test) and desensitization time constant of ASICs (p = 0.96, paired t-test) in these outside-out patches (n = 4). The current amplitude of ASICs were 61±19 pA, 65±21 pA and 67±22 pA before, during and after GABA application, respectively. The desensitization time constants of ASICs were 972±120 ms, 962±62 ms and 897±43 ms before, during and after GABA application, respectively. (TIF) [file pone.0021970.s004.tif]

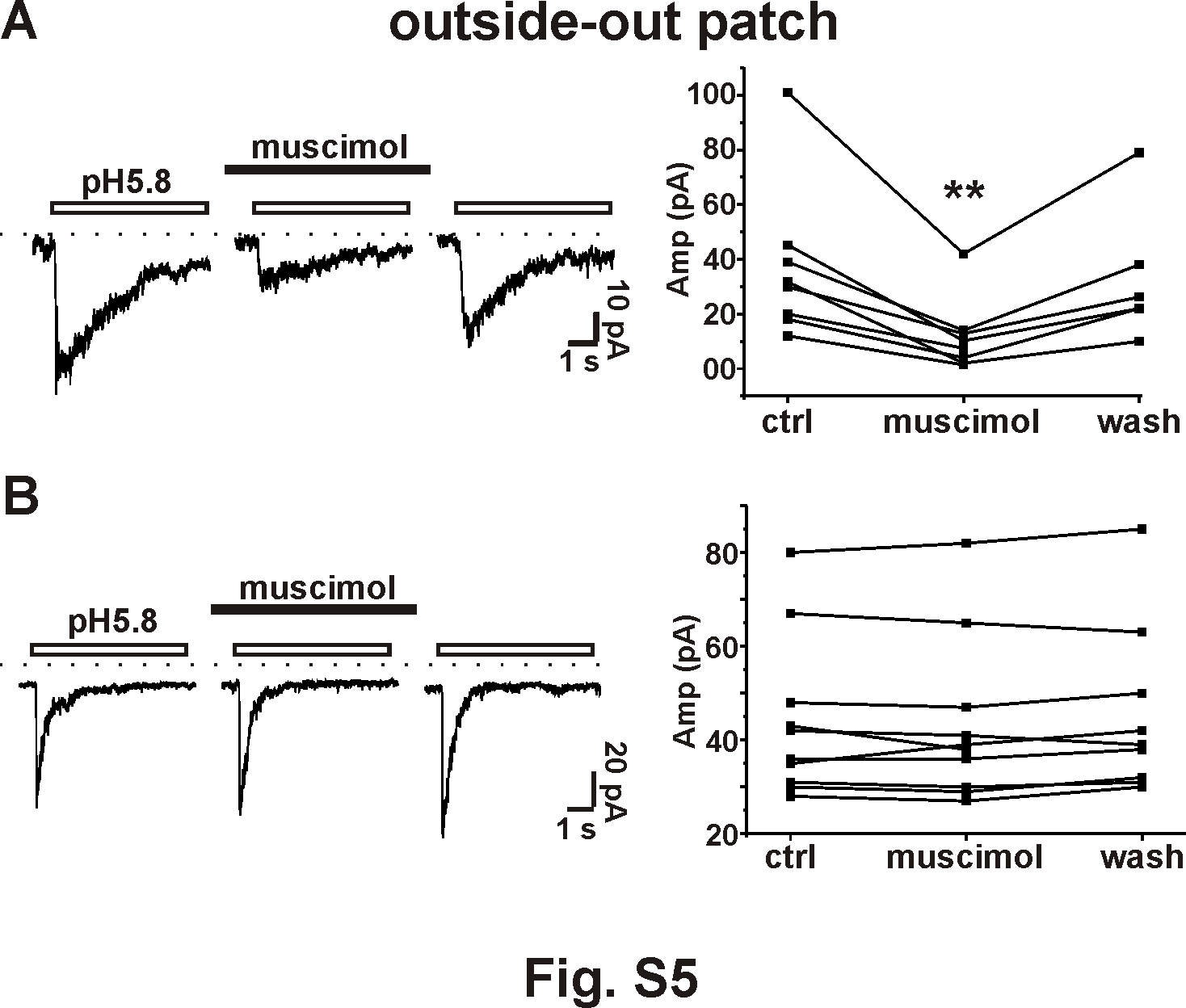

Supplement: Figure S5 — Muscimol modulates ASICs in some recordings (A) but not in other recordings (B) in outside-out patches. A, left, representative traces of ASIC currents in the absence or presence of muscimol; right, bar graph showing the peak amplitude of ASIC currents with or without muscimol. In these outside-out patch recordings (n = 8), muscimol markedly (p<0.01, paired t-test) decreased the current amplitude of ASICs from 37±10 pA to 12±5 pA, which were recovered to 33±9 pA after washout of muscimol. B, left, representative traces of ASIC currents in the absence or presence of muscimol; right, bar graph showing the peak amplitude of ASIC currents. In these recordings, muscimol did not (p = 0.44, paired t-test) affect the current amplitude of ASICs (n = 10). The current amplitude of ASICs were 44±5 pA, 43±6 pA and 47±6 pA before, during and after muscimol application, respectively. (TIF) [file pone.0021970.s005.tif]

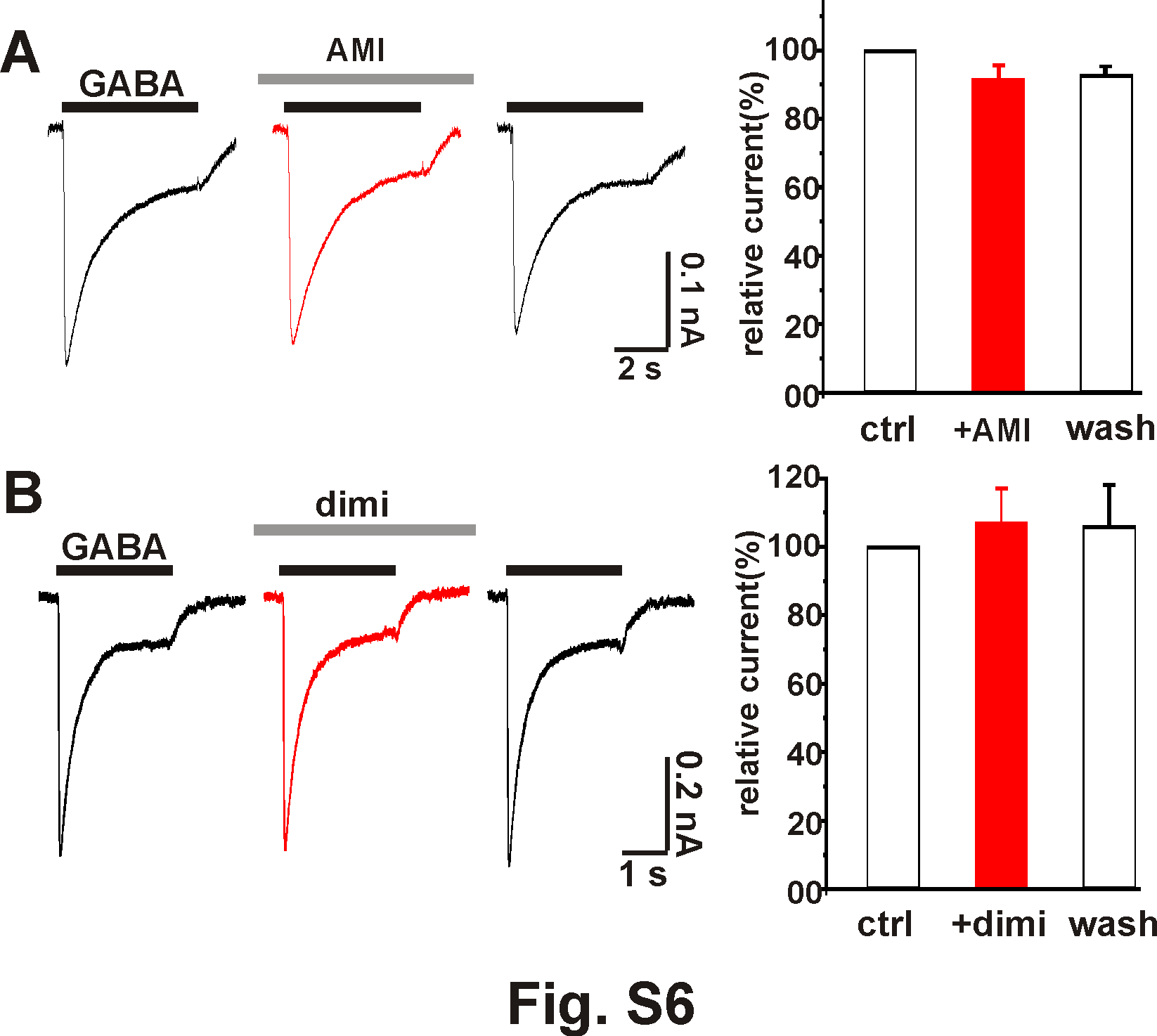

Supplement: Figure S6 — Amiloride and diminazene do not block GABAA receptors. A left, representative traces of GABA-evoked currents (100 µM) in the absence (black) or presence (red) of amiloride (AMI, 200 µM). Right, bar graph showing the relative GABA-current in the absence or presence of amiloride. Amiloride did not (p = 0.22, paired t-test; n = 4) affect GABAA receptors. The relative peak amplitudes of GABA-currents were 0.91±0.04 and 0.91±0.02 during amiloride application and after washout of amiloride, respectively. B left, representative traces of GABA-currents in the absence (black) or presence (red) of diminazene (dimi, 50 µM). Right, bar graph showing the relative GABA-current with or without diminazene. p = 0.63, paired t-test, n = 5. The relative peak amplitudes of GABA-currents were 1.07±0.10 and 1.06±0.11 during diminazene application and after washout of diminazene, respectively. (TIF) [file pone.0021970.s006.tif]

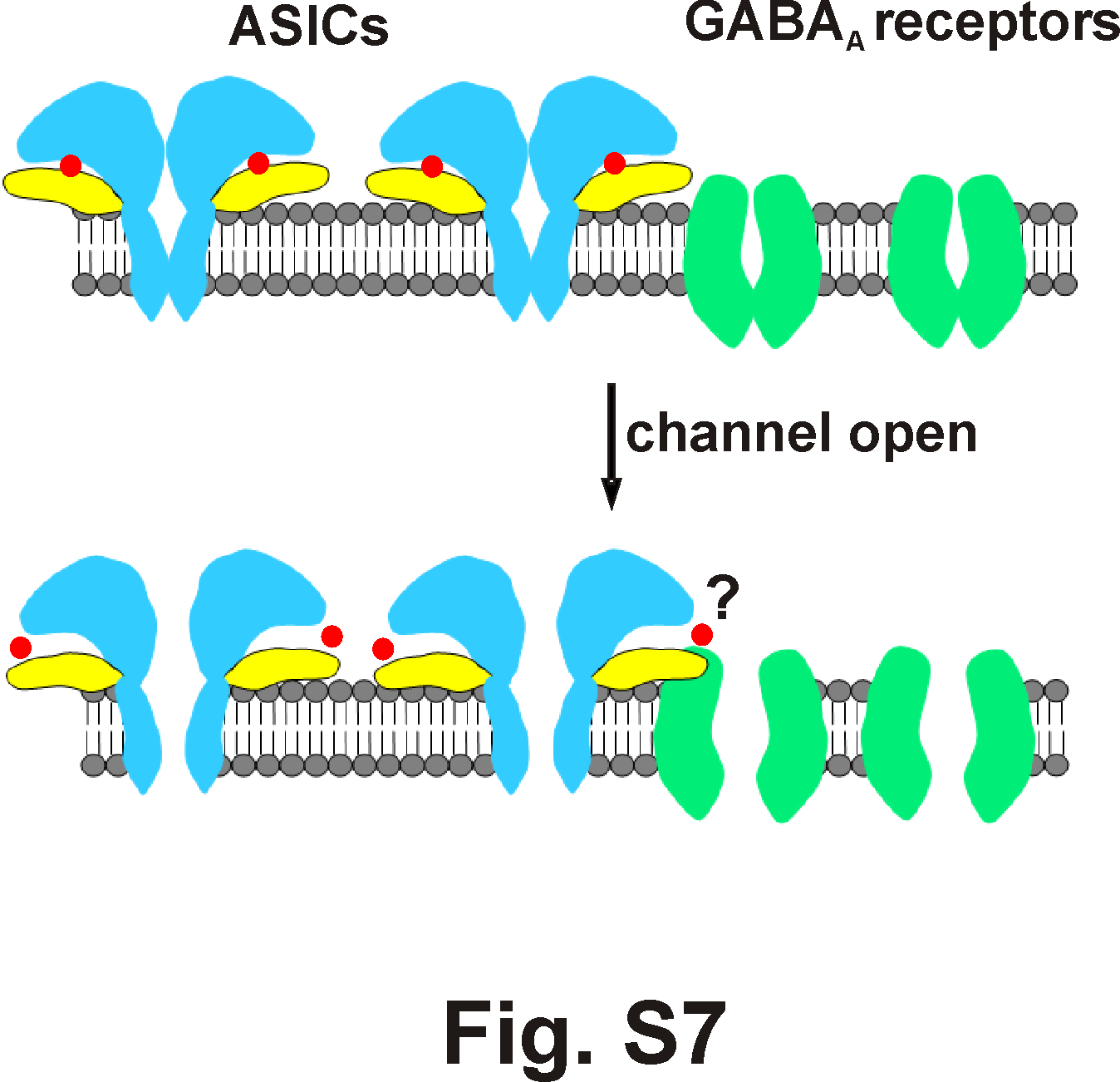

Supplement: Figure S7 — A working model of interaction of ASICs with GABAA receptors. In the resting states (top), GABAA receptors do not intervene with the function of ASICs. When GABAA receptors are opened (bottom), part of receptors that locate close to ASICs may interact with the open ASICs and thereby modify ASIC functions. This interaction may be conformation-dependent. It is unknown whether the chloride ions (red circles) bound in the extracellular thumb domains of ASICs participate in the interaction. GABAA receptors may not affect ASICs if two receptors locate distantly. (TIF) [file pone.0021970.s007.tif]
